# Spontaneous membrane-translocating peptides: influence of peptide self-aggregation and cargo polarity

**DOI:** 10.1038/srep16914

**Published:** 2015-11-16

**Authors:** Sara Macchi, Giovanni Signore, Claudia Boccardi, Carmine Di Rienzo, Fabio Beltram, Francesco Cardarelli

**Affiliations:** 1 NEST, Scuola Normale Superiore and Istituto Nanoscienze-CNR, Piazza San Silvestro 12-56127 Pisa, Italy; 2 Center for Nanotechnology Innovation @NEST, Istituto Italiano di Tecnologia, Piazza San Silvestro 12-56127 Pisa, Italy

## Abstract

Peptides that translocate spontaneously across cell membranes could transform the field of drug delivery by enabling the transport of otherwise membrane-impermeant molecules into cells. In this regard, a 9-aminoacid-long motif (representative sequence: **PLIYLRLLR**, hereafter Translocating Motif 9, TM9) that spontaneously translocates across membranes while carrying a polar dye was recently identified by high-throughput screening. Here we investigate its transport properties by a combination of *in cuvette* physico-chemical assays, rational mutagenesis, live-cell confocal imaging and fluorescence correlation spectroscopy measurements. We unveil TM9 ability to self-aggregate in a concentration-dependent manner and demonstrate that peptide self-aggregation is a necessary –yet not sufficient– step for effective membrane translocation. Furthermore we show that membrane crossing can occur with apolar payloads while it is completely inhibited by polar ones. These findings are discussed and compared to previous reports. The present results impose a careful rethinking of this class of sequences as direct-translocation vectors suitable for delivery purposes.

The ability to cross the plasma-membrane and gain access to the cell interior, often even to a specific subcellular compartment, is a requirement for any intracellular drug-delivery protocol. To this end, *direct* membrane crossing is a particularly attractive approach to cytoplasmic delivery since it avoids the complications of vesicle-mediated internalization pathways^1^,^2^,^3^,^4^,^5^,^6^. This is still an unsolved issue, however, but today, peptide-based strategies appear to be particularly promising. Indeed, high-delivery yields, low toxicity, wide range of target cells, and the possibility to easily modify their sequence/function make peptides an excellent platform for the engineering of efficient transporters. Of note in the field, Marks and co-workers recently developed a straightforward combinatorial chemistry approach capable of achieving the high-throughput screening of thousands of sequences at the same time^7^. These authors selected out of several thousand sequences 18 Spontaneous Membrane-Translocating Peptides (SMTPs) with a characteristic length of 12 amino acids and demonstrated their ability to deliver low-molecular-weight polar cargo-molecules across synthetic lipid bilayers and into live cells at relatively low peptide concentration (0.5–2 μM). Importantly these peptides were shown not to irreversibly affect membrane integrity. A number of later studies (both theoretical and experimental) were performed on one of these representative peptides (known as Translocating Peptide 2, TP2; sequence: **PLIYLRLLR**-G-QF) that provide detailed knowledge on the mechanism yielding its spontaneous membrane translocation^8^,^9^,^10^,^11^,^12^,^13^,^14^. Notably, available data clearly ascribe TP2 translocating activity to the very first 9 residues of its sequence (highlighted in bold above), while the additional 3 residues were only functional to specific *in-cuvette* translocation assays (for details see Ref. 7). Yet, no detailed reports are available so far on the properties of the putative 9-aa translocating motif of TP2. Here we address this matter by a combination of *in cuvette* physico-chemical assays, live-cell confocal imaging, and fluorescence correlation spectroscopy measurements on fluorescently-labeled variants of this 9-aa peptide, hereafter named Translocating Motif 9 (TM9). We use ATTO 425 and Tetramethylrhodamine-5-maleimide (TAMRA) as representative apolar and polar low-molecular-weight cargoes, respectively. By means of the standard “pyrene 1:3 ratio method”^15^, we demonstrate that TM9 is able to self-aggregate in a concentration-dependent manner, regardless of the polarity of the attached cargo. In particular, we identify the Critical Micelle Concentration (CMC)^16^ above which the peptide appears in an aggregated form. We confirm this result by Dynamic Light Scattering (DLS) measurements and find that the aggregated form corresponds to a nanoparticle with a hydrodynamic radius of about 100–150-nm. Concentration-dependent aggregation and cargo-polarity together determine the peptide translocation properties in live cells. In particular, while TAMRA-labeled TM9 enters cells by a vesicle-mediated process at any tested concentration, its ATTO 425-labeled counterpart is able to effectively relocate into cells by direct plasma-membrane translocation. In this latter case, however, we additionally prove that direct translocation switches-on only above the peptide characteristic CMC, while endocytosis dominates below the CMC. Furthermore, as revealed by quantitative fluorescence microscopy on live cells, translocation of the TM9-ATTO 425-based nanoparticle occurs preferentially across selected plasma membrane regions, analogously to what already observed for several cell-penetrating peptides^17^. In addition, our data suggest a critical role of integer extracellular glycosaminoglycans (GAGs) for this localized process to be effective, as GAGs enzymatic digestion leads to peptide homogeneous translocation throughout the plasma membrane. Based on this knowledge, we finally propose a direct comparison of TM9 with its precursor sequence, TP2, widely used in the literature. We show that TP2 is able to form aggregates in a concentration-dependent manner, similarly to TM9. This behavior, however, does not correspond to the expected concentration-dependent translocation of TP2 into cells, since endocytosis is shown to be the dominant mechanism of entry under the experimental conditions tested here, regardless of the specific fluorophore-cargo chosen. Importantly, we discuss this apparent contradiction and relate it to the specific experimental conditions that significantly affect the peptide physicochemical behavior and, as a consequence, its membrane translocation ability. Collectively, our results impose a careful rethinking of this class of sequences as direct-translocation vectors for delivery purposes. We believe that peptide propensity to self-aggregate, exact peptide sequence, and nature of the attached cargo need further attention and study in an effort to rationally engineer a new generation of spontaneously-translocating sequences.

## Results and Discussion

### TM9 direct translocation across the plasma membrane depends on the polarity of the cargo and on peptide concentration

**Figure 1:**
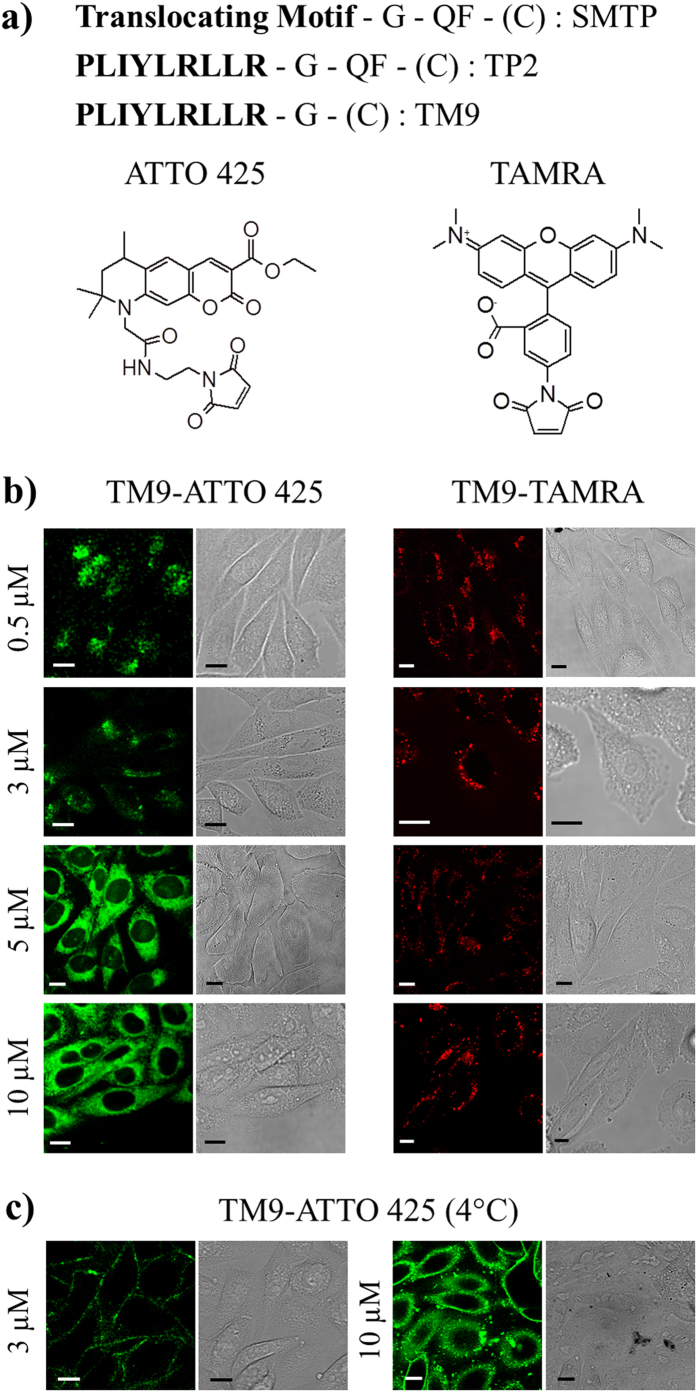
SMTP general structure, TP2 and derived TM9 peptide sequences, fluorophores studied here and cell uptake of dye-labeled TM9. **(a**) SMTP structural composition, TP2 precursor sequence as representative example and TM9 peptide sequence with ATTO 425 and TAMRA structures. (**b**) Confocal images of cells treated with increasing concentrations of TM9-ATTO 425 and TM9-TAMRA in free serum medium. Concerning the former, endocytic bright spots are detectable at 0.5 and 3 μM, while direct translocation is prevalent at 5 and 10 μM. Concerning the latter, only endocytosis is present at all the concentrations tested. Scale bars: 10 μm. (**c**) Confocal images of cells treated with 3 μM (left) and 10 μM (right) of TM9-ATTO 425 at 4 °C. While in the former case only cell plasma membranes are labelled (i.e. endocytosis is blocked), in the latter one diffuse cytoplasmic staining is still evident (i.e. direct translocation occurs).

**Figure 2:**
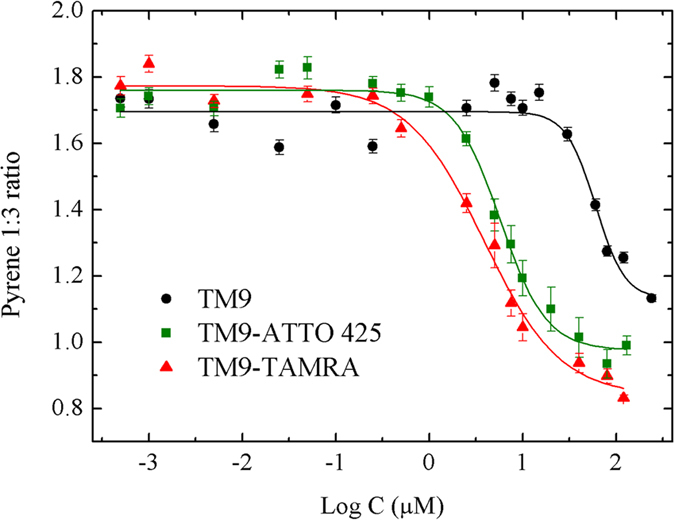
Variation of the pyrene 1:3 ratio at increasing concentrations of unlabeled, ATTO 425- and TAMRA- labeled TM9 peptide. Vertical bars: standard errors.

**Figure 3:**
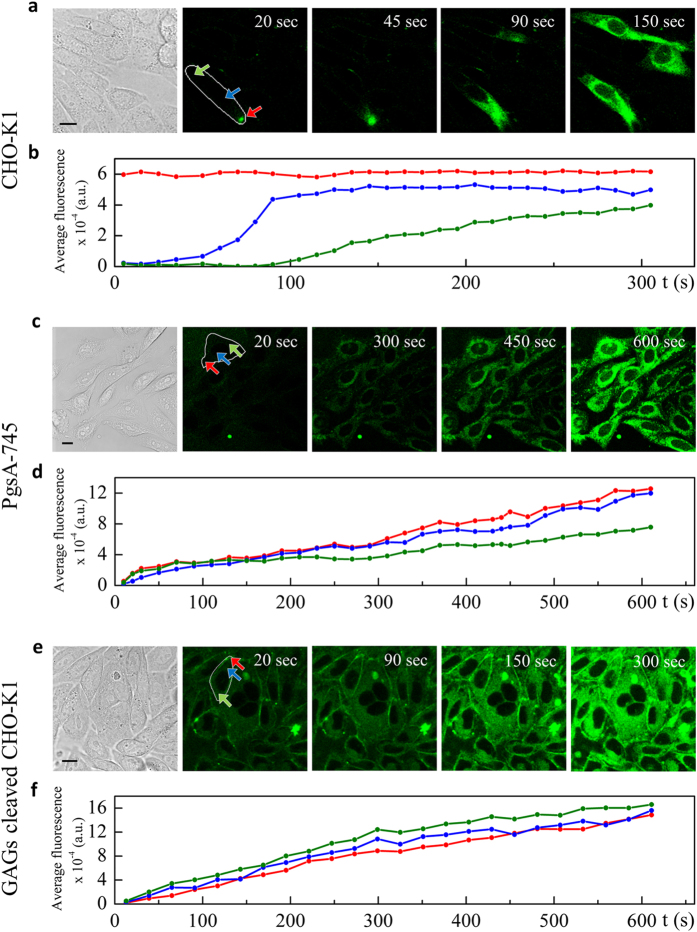
Cell uptake and diffusion kinetics of direct translocating TM9-ATTO 425 in GAGs rich and deficient cells. (**a**,**c**,**e**) diffusion of TM9-ATTO 425 solutions 12 μM in CHO-K1, 10 μM in PgsA-745 and 10 μM in GAGs cleaved CHO-K1, respectively. A NZ-dependent uptake is clearly detectable in the former case, while a homogeneous diffusion of peptide is shown for GAG-deficient and GAG-cleaved cells. The colored arrows indicate the ROIs used to evaluate peptide diffusion. (**b**,**d**,**f**) plot of the average fluorescence (AU) with respect to time (s) in the selected ROIs. In the case of CHO-K1 a different trend is shown with respect to the location within the cell, while, a linear increase occurs in all the locations within PgsA-745 and GAG-cleaved cells. Scale bars: 10 μm.

**Figure 4:**
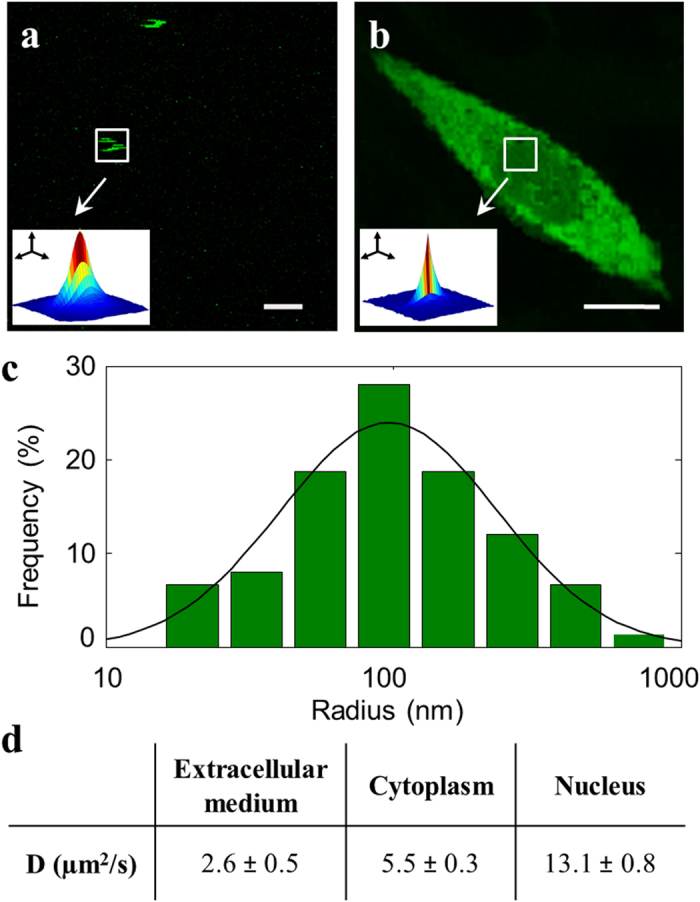
RICS and single-particle RICS analysis and results for TM9-ATTO 425. (**a**) Nanoparticle aggregates found in the external solution, for which RICS measurement was repeated overall 30 times. Scale bar: 1.5 μm. (**b**) A cell region selected for RICS experiments. Scale bar: 10 μm. (**c**) Size distribution and gaussian fitting for nanoparticles found in the external solution. The mean radius is 100 ± 30 nm. (**d**) TM9-ATTO 425 diffusion coefficients (μm^2^/s) obtained from RICS for nanoparticles and for internalized peptide.

**Figure 5:**
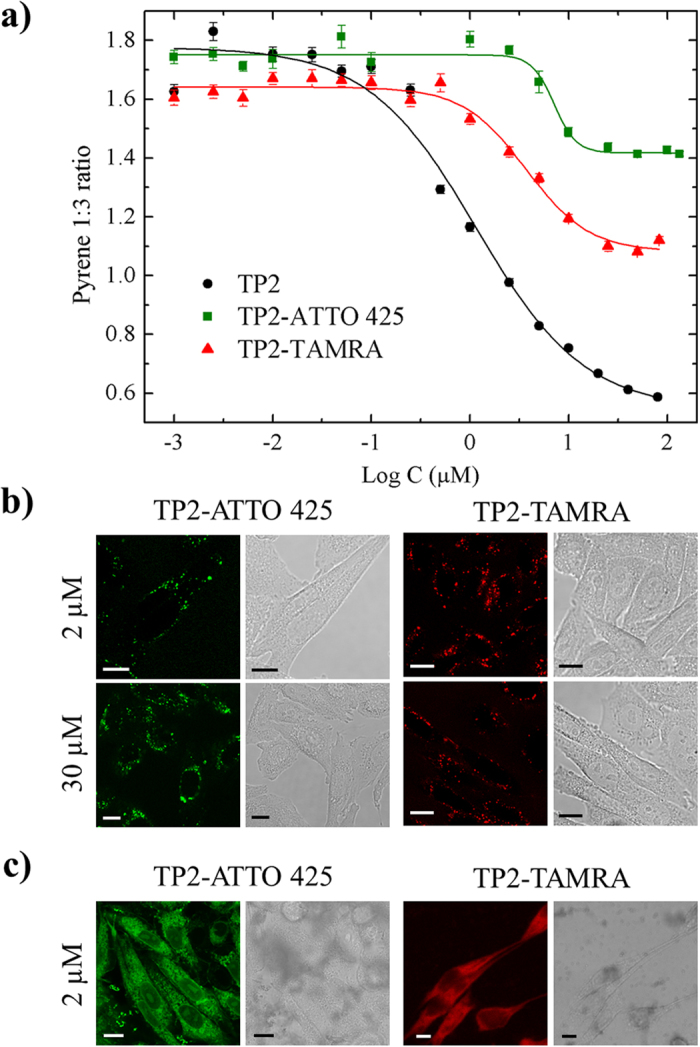
Decreasing of pyrene 1:3 ratio at TP2 increasing concentrations, TP2 cell uptake and cell uptake before and after the homogenization of TP2 solution (1% DMSO). (**a**) Plot of the pyrene 1:3 ratio of free and dye-labeled TP2 peptide. Vertical bars: standard errors. (**b**) Confocal images of cells treated with increasing concentrations of TP2-ATTO 425 and TP2-TAMRA in free serum medium. Scale bars: 10 μm. (**c**) Confocal images of cells treated with TP2-ATTO 425 and TP2-TAMRA at a final concentration of 2 μM (peptide added to the cell medium directly from the DMSO stock solution). Scale bars: 10 μm.

Marks and co-workers recently introduced a straightforward combinatorial peptide chemistry and orthogonal high-throughput screening to select peptides that spontaneously translocate across synthetic lipid bilayer membranes without permeabilization effects, while carrying a small polar cargo (TAMRA)^7^. A family of Spontaneous Membrane Translocating Peptide (SMTP) sequences with the general structure reported in Fig. 1a was identified. In particular, the first 9 residues represent the screened segment, with putative translocating properties (example: ^1^**PLIYLRLLR**^9^, as in Fig. 1a). A Glycine in position 10 constitutes a flexible linker, while Glutamate in position 11 and Phenylalanine in position 12 are functional to the *in vitro* test of membrane permeability used^7^. A C-terminal Cysteine residue was also added for labeling purposes. Following this general strategy, and in order to better evaluate the properties of the putative 9-aa-long translocating motif, we removed the Glutamate and Phenylalanine residues, while preserving the Glycine as a flexible linker and Cysteine to chemically attach a cargo moiety (overall sequence: **PLIYLRLLR**-G-C, hereafter named Translocating Motif 9, TM9). The purified peptide was labeled on the Cys residue with either ATTO 425 (as model of apolar cargo) or TAMRA (as a model of polar cargo, in analogy with previous studies^7^,^13^,^14^) (Fig. 1a). Labeled peptides were tested on live CHO-K1 cells by confocal fluorescence microscopy. As a first test, we performed experiments on cells exposed to increasing concentrations of either TM9-ATTO 425 or TM9-TAMRA. As detailed in the *Experimental Procedures* section, purified dye-labeled peptides were pre-dissolved in water solution, sonicated, quantified by UV-Vis absorbance, and added to the cell medium at the desired concentration (in both cases, fluorescent peptides are incubated with cells for 20 minutes before washing). As reported in Fig. 1b, we observed that for TM9-ATTO 425 concentrations up to 3 μM only fluorescent vesicles (bright spots in Fig. 1b-0.5 and 3 μM) are detected, indicating that endocytosis is the dominant mechanism responsible of peptide uptake. On the contrary, an intense and diffuse fluorescence signal is present at higher peptide concentrations (e.g. 5 and 10 μM), suggesting that a vesicle-independent direct translocation process across the plasma membrane becomes dominant in this regime (Fig. 1b-5 and 10 μM). The observed fluorescence staining (labeled cytoplasm and apparently dark nucleus) is consistent with the peculiar spectral properties of ATTO 425 (Fig. S1): just like many coumarin derivatives^18^, in fact, this fluorophore markedly increases its fluorescence quantum yield in apolar, lipophilic environments of the cell (cytoplasm) compared to aqueous environments (nucleus and surrounding medium). By contrast, under the same experimental conditions, TM9-TAMRA clearly shows punctuate fluorescence throughout the complete range of tested concentrations, with no apparent direct translocation into the intracellular medium (Fig. 1b). To reinforce our conclusion that the massive cytoplasmic staining observed for TM9-ATTO 425 at high concentrations depends on direct plasma membrane translocation we performed an uptake experiment at 4 °C, a well-known strategy to block active endocytosis while preserving direct permeation^5^. As can be seen in Fig. 1c, endocytosis of TM9-ATTO 425 at 3 μM is almost completely inhibited (i.e. only accumulation of the peptide at the level of the plasma membrane can be detected, Fig. 1c, left panels). On the contrary, under the same experimental conditions, TM9-ATTO 425 at 10 μM still shows diffuse cytoplasmic staining, thus corroborating the hypothesis of a temperature-independent direct translocation process across the membrane. To exclude any possible toxic effect of the labeled peptides on cell viability, we incubated cells for 2 hours in presence of varying peptide concentrations and performed the standard WST-8 assay 24 hours after removal of the peptide solution (Fig. S2).

### TM9 is able to self-aggregate in a concentration-dependent manner

The translocation properties observed for TM9-ATTO 425 prompted us to speculate that peptide physicochemical properties (e.g. aggregation state) may change in a concentration-dependent manner, thus affecting transport properties in cells. As a first test, we dissolved either unlabeled or dye-labeled peptides in water solutions and performed a standard pyrene 1:3 ratio experiment, as a means to test their aggregation propensity^15^. Notably (Fig. 2), a significant decrease of the 1:3 ratio is detected going from 0.5 μM to 100 μM peptide concentration in all cases: a clear flex at a threshold CMC value is identified, analogously to previous measurements on short peptide sequences^19^,^20^. The CMC values for TM9, TM9-ATTO 425 and TM9-TAMRA are shown in Table 1. It is worth noting that the presence of a fluorophore, regardless of its polarity, lowers the CMC value of about one order of magnitude.We measured 58 ± 4 μM for CMC in the case of unlabeled TM9 and 5.7 ± 0.8 μM and 3.8 ± 0.5 μM for its ATTO 425- and TAMRA-labeled variants, respectively. This effect prompted us to test the intrinsic propensity of isolated fluorophores to form aggregates: we found that both TAMRA and ATTO 425 have a characteristic CMC in the μM range, even if at higher values (∼80 μM for TAMRA and ~25 μM for ATTO 425, see Fig. S3), in line with available data^21^. In order to evaluate the dimension of the peptide aggregates we performed Dynamic Light Scattering (DLS) measurements at concentrations above the CMC threshold. Table 1 shows the measured hydrodynamic radii for unlabeled and dye-labeled TM9 nanoparticles.

### Addressing the spatiotemporal regulation of TM9 translocation in live cells

In order to get further insight on peptide translocation into cells we incubated CHO-K1 cells in serum-free medium, added the selected concentration of TM9-ATTO 425 (see also the *Experimental Procedures* section) and performed a time-lapse, real-time imaging of peptide internalization process, this time without replacing the cell medium (Supporting Information, Movie 1). This latter procedure, made possible by the intrinsic spectral properties of ATTO 425 (as discussed above), allowed us to image the otherwise elusive process of peptide entry into cells. Interestingly, in fact, at concentrations above the CMC, few micrometric zones (typically one or two per cell) in which the fluorescence signal suddenly increases (i.e. the peptide locally concentrates) are detected on the plasma membrane shortly after peptide administration. Then fluorescence rapidly diffuses into the rest of the cell (Fig. 3a). Accordingly, if the average fluorescence intensity is measured in time at different locations within the cell (e.g. arrows in Fig. 3a) a quantitative picture of the timing of peptide intracellular spreading can be achieved. As reported in the plot of Fig. 3b, a nearly-constant intensity value is obtained if the ROI is placed at the peptide entry point, while a time lag of null fluorescence intensity is measured if the ROI is far from the entry point (with time lag proportional to the distance from the entry point). Overall, about 50% of the cells are interested by the translocation process. Of note, in a number of reports similar translocation processes originating from spatially-restricted sites of the plasma membrane (also known as “Nucleation Zones”, NZs) were related to peptide binding to selected extracellular-matrix components and subsequent translocation into the intracellular medium^17^,^22^,^23^,^24^. Here we test the hypothesis that TM9 uptake and translocation might be regulated also at the extracellular level. In this regard, a classical molecular target is represented by glycosaminoglycans (GAGs) that, together with proteins, constitute the “glycocalyx” that covers cell surface. To test the possible role of GAGs we performed a confocal microscopy experiment using PgsA-745 cells. This cell line, derived from CHO-K1 cells, does not produce GAGs, as it does not express xylosyltransferase (a required enzyme in GAG synthesis)^25^,^26^. We administered TM9-ATTO 425 at 10 μM to PgsA-745 and performed a time-lapse acquisition (see also Supporting Information, Movie 2). We observed that, differently from wild type CHO-K1 cells, the peptide enters nearly 100% of the cells by a translocation process that is homogeneously distributed throughout the plasmatic membrane (i.e. no NZs are detected, Fig. 3c). This is confirmed by the spatial analysis of peptide uptake in different locations within the cell (i.e. a linear increase of fluorescence in all the selected ROIs is detected, with no time lag, Fig. 3d). As a last control experiment, we analyzed peptide entry into CHO-K1 cells in which the GAGs were enzymatically digested by chondroitinase ABC (ChABC, Fig. S4). Upon administration of TM9-ATTO 425 at 10 μM concentration in serum-free medium, we monitored internalization by real-time imaging (Supporting Information, Movie 3). We detected homogeneous spreading of the fluorescence signal in 100% of the cells (Fig. 3e); fluorescence increased steadily during the observation time, with no time lag (Fig. 3f). Collectively, these results point at a significant role of integer GAGs in re-directing the peptide entry process towards spatially-restricted sites of the plasma membrane or NZs. Based on the present data and on the well-recognized role of GAGs as endocytosis effectors^27^,^28^,^29^, we cannot exclude that NZs are sites of GAG absence (or marked spatial inhomogeneity) rather than GAG accumulation.

In order to further probe the spatiotemporal regulation of TM9-ATTO 425 translocation, we analyzed its extra- and intracellular diffusion properties by Raster Image Correlation Spectroscopy (RICS)^30^ measurements. These measurements were performed at a peptide concentration above the CMC (10 μM in the case reported in Fig. 4). Concerning the external solution, in some of the collected images we were able to identify bright diffusing spots blurred by the scanning process (Fig. 4a): these bright spots likely correspond to peptide nano-aggregates. By applying a single-particle RICS protocol (see *Experimental Procedures*) we retrieved a diffusion coefficient of 2.6 ± 0.5 μm^2^/s (Fig. 4d), compatible with aggregates with a hydrodynamic radius of about 100 nm (in good agreement with DLS results) (Fig. 4c). In order to identify the aggregation state of TM9-ATTO 425 within the cell, we performed the same RICS measurement in the cytoplasm and in the nucleus (Fig. 4b). Here, we were not able to identify isolated bright diffusing spots and we found a diffusion coefficient of 5.5 ± 0.3 μm^2^/s in the cytoplasm and of 13.1 ± 0.8 μm^2^/s in the nucleus (Fig. 4d). This suggests a disruption of peptide aggregates during or after cell permeation. In fact, the measured diffusion coefficient in live cell is in agreement with previous intracellular measurements on analogous monomeric peptides^31^,^32^. Moreover, the twofold lower diffusion coefficient measured in the cytoplasm compared to the nucleus indicates a higher propensity of the peptide to interact with the cytoplasmic environment. Please note that this result is somewhat expected given the presence of membranous structures within the cytoplasm only.

### Comparison of TM9 with the TP2 precursor sequence

At this point, one must check whether the properties unveiled so far for TM9 are shared by its precursor sequence, TP2. To this end, we performed the pyrene 1:3 ratio assay on TP2 dissolved in water at varying concentrations, ranging from 0.5 μM to 100 μM (Fig. 5a). Notably, the calculated I1/I3 parameter varies non-linearly with peptide concentration, as expected for a concentration-dependent aggregation process. In this case, and in contrast to TM9, the characteristic CMC value of the unlabeled peptide (1.0 ± 0.2 μM) increases upon addition of the fluorophore, regardless of fluorophore polarity (CMC = 7.1 ± 0.9 μM in the case of ATTO 425, and CMC = 3.7 ± 0.5 μM in the case of TAMRA, see also Table 1). As expected, DLS measurements confirm the presence of peptide-based aggregates at concentrations above the CMC for both unlabeled and labeled TP2 and yield an estimate of the aggregate size (about 160 nm for TP2 and about 110 nm for both types of dye-labeled TP2). We then investigated cell uptake of the two fluorescent variants of TP2 at concentrations both below and above their respective CMC values (Fig. 5b). Quite surprisingly, no direct translocation was observed for TP2, regardless of the selected concentration and of the chosen fluorescent cargo (polar or apolar). In fact, as shown by confocal images in Fig. 5b, only punctuate fluorescence staining was detected within live cells after peptide administration, suggesting endocytosis as the dominant uptake mechanism for these TP2 adducts.These results do not appear to be consistent with previous reports on TP2^7^,^14^ and this, in turn, suggests the need for a careful comparison of the experimental protocols adopted. For what concerns *in vitro* cell studies, in previous reports dye-labeled TP2 was added from stock DMSO solutions directly to the cell medium up to the desired final concentration^7^,^14^. Indeed, if we adopt this same procedure, we do obtain direct translocation for both TP2-ATTO 425 and TP2-TAMRA even below the CMC value (see, for instance, the behavior of both TP2-ATTO 425 and TP2-TAMRA at 2 μM reported in Fig. 5c). Unfortunately, although not affecting cell viability (its final concentration in the cell medium is always around 1% or even less, as in the literature), DMSO can influence TP2 solubilization by altering peptide/water miscibility kinetics and lead to the formation of precipitates (see *in cuvette* absorption measurements reported in Fig. S5). As a consequence, this type of protocol does not appear to be transferable from these *in vitro* tests to more complex situations like tissues or entire organisms.

## Conclusions

In this work we addressed the spontaneous membrane-translocation properties of a recently-introduced class of short peptidic sequences (representative translocating motif: **PLIYLRLLR**, TM9). Thanks to a combination of *in cuvette* physico-chemical assays, rational mutagenesis, and live-cell fluorescence-based measurements we demonstrated that TM9 self-aggregates in a concentration-dependent manner.Moreover we showed that self-aggregation is a necessary –but not sufficient– step for effective membrane translocation. Indeed, membrane crossing is enabled by apolar payloads while it is inhibited by polar ones. These new results on this interesting class of sequences show the need of further studies for their exploitation as direct-translocation vectors suitable for drug-delivery purposes. In particular, peptide propensity to self-aggregate, peptide sequence and influence of the attached cargo must be considered as key parameters in the effort of rationally engineer new spontaneously-translocating sequences, better tailored to delivery applications both *in vitro* and *in vivo*.

### Experimental procedures

#### Peptide synthesis, purification and labeling

All peptides were prepared by solid-phase synthesis using Fmoc chemistry on an automatic Liberty Blue Peptide Synthesizer with an integrated microwave system (CEM, North Carolina, USA). HPLC analysis and purification were performed on a Dionex Ultimate 3000 PLC system with autosampler. Crude peptides were purified by RP-HPLC on a Jupiter 4 μm Proteo 90 Å column (250 × 10 mm; Phenomenex) using these solvents: water:TFA 100:0.01 v/v (eluent A)/acetonitrile:water:TFA 95:5:0.01 v/v (eluent B), flux 5 ml/min. The identity of purified product was confirmed by electrospray mass spectroscopy, using an API3200QTRAP Hybrid Triple Quadrupole/Linear Ion Trap (ABSciex, Foster City, California, USA). Labeling of purified peptides was performed as follows: a suitable amount of peptide (10 μmol) was dissolved in 0.2 mL of freshly degassed 10 mM PBS buffer (pH 7.4). 1.5 equivalents of fluorophore, ATTO 425-maleimide (ATTO-TEC GmbH, Germany) or Tetramethylrhodamine-5-maleimide (TAMRA, Sigma Aldrich), dissolved in DMF were added to the solution. The mixture was reacted for 2 h at 25 °C. Crude product was purified by HPLC and the identity of labeled peptides was confirmed by LC-MS. The purified product was freeze-dried and stored at −80 °C. When needed, it was dissolved in water and the concentration of each peptide stock solution was verified by UV–vis absorbance (Jasco 550 spectrometer, Jasco, Tokyo, Japan).

### Preparation of peptide solution for translocation experiments

Homogeneous peptide solutions used for translocation experiments were always freshly prepared. Purified dye-labeled peptides were pre-dissolved in 100–200 μl of water and sonicated for 15 minutes at 25 °C. Then they were quantified by UV-Vis absorbance (concentration ranging between 100 and 200 μM; for ATTO 425 Maleimide: ε_436_(water) = 45000 cm^−1^M^−1^, for TAMRA: ε_543_(water) = 80000 cm^−1^M^−1^). An appropriate amount from this stock water-solution was diluted in 200–300 μl of serum-free DMEM F-12 growth medium and then administered to cells. Ionic strength was adjusted to physiological values adding the required amount of 10X PBS buffer.

### Cell culture and translocation experiments

Chinese Hamster Ovary (CHO-K1) and PgsA-745 cells were purchased from ATCC (reference numbers: CCL-61 and CRL-2242, respectively) and grown in DMEM F-12 medium supplemented with 10% of Fetal Bovine Serum (FBS) at 37 °C with 5% CO_2_, according to manufacturer’s instructions. In order to perform translocation experiments, cells were plated onto 35-mm glass-bottom petri dishes (WillCo-dish GWSt-3512) 24 h before the experiment. The day of the experiment, cells were washed with PBS at room temperature. At this point the peptide solution was homogeneously added, and cells incubated for 20 minutes at 37 °C. In standard uptake experiments, after incubation, cells were washed with PBS and fresh complete medium was added before confocal imaging. For the experiment of real-time imaging of peptide direct translocation through nucleation zones (limited to TM9-ATTO 425 at high concentration) we did not wash the peptide solution before imaging, for the reasons explained in the Results section.

### Confocal microscopy and Raster Image Correlation Spectroscopy (RICS)

Confocal imaging experiments were performed with a Leica TCS SP5 SMD inverted confocal microscope (Leica Microsystems) interfaced with a diode laser (Picoquant) for excitation at 405 nm. Glass-bottom Petri dishes containing plated cells were mounted in a temperature-controlled chamber at 37 °C and 5% CO_2_ (Leica Microsystems) and viewed with a 63 × 1.2 numerical aperture (NA) water immersion objective (Leica Microsystems). The excitation wavelengths were 405 nm and 561 nm for ATTO 425 and TAMRA, respectively. The collection range adopted was 450–550 nm and 580–680 nm for acquiring ATTO 425 and TAMRA, respectively. The diameter of the detection pinhole was set to the size of 1 Airy disk. All images and videos collected were analyzed by ImageJ software version 1.440 (NIH Image; http://rsbweb.nih.gov/ij/). RICS measurements were performed with an Olympus FluoView 1000-ASW-2.0 confocal laser scanning microscope using a 60× (NA 1.2) planApo water-immersion objective. The diameter of the detection pinhole was set to the size of 1 Airy disk. All experiments were carried out at 37 °C and 5% CO_2_ using an incubation chamber enclosing the microscope stage and body. Sequential image series at 12 bits were collected at a fixed pixel size of 50 nm and with a pixel dwell time of 10 μs for measurements in solution and in cells. Typically, a ROI of 256 × 256 pixels (corresponding to an area of 6.4 × 6.4 μm) was selected and 200 total frames were collected. RICS correlation function was calculated by the SimFCS software, developed at the Laboratory for Fluorescence Dynamics (www.lfd.uci.edu). Fitting of RICS function to extract the diffusion coefficient (D, μm^2^/s) of the dye-labeled peptide was performed by SimFCS software. In order to measure the diffusion coefficient of the peptide nanoparticles directly in the media above the cells we applied the following protocol (also named ‘single-particle RICS’ in the main text in order to distinguish it from the classical RICS analysis). Peptide nanoparticles appear as bright isolated spots during the imaging process. Since they are diffusing, their image is blurred by the raster scan process. The RICS principle can be thus used to estimate their diffusivity. In this case, the signal-to-noise ratio is so high that averaging over many frames is not required. Thus, we calculate the RICS correlation function in a ROI containing each selected particle separately and measure the diffusion coefficient one particle at a time by fitting with a free diffusion model^33^,^34^. This protocol allows obtaining an estimate of the hydrodynamic radius of the diffusing particle, analogously to the DLS measurement but through direct observation of a single particle.

### GAGs labeling and cleavage in CHO-K1 cells

Fluorescein-labeled *Wisteria Fluribunda* Lectin (2 mg/ml, Sigma Aldrich, catalog number: FL-1351) was used as a well-recognized marker of GAGs. It was diluted 1:100 in DMEM F-12 (10% FBS) and added to CHO-K1 cells, which were then incubated for 2 hours at 37 °C with 5% CO_2_. Chondroitinase ABC (Sigma Aldrich) was then added at a final concentration of 0.2 U/ml obtaining an efficient cleavage of surface proteoglycans. After 4 hours of incubation, translocation experiments were performed, as described above.

### WST-8 Cell Viability Assay

Cytotoxicity of unlabeled peptides and dye-labeled peptides was evaluated by the WST-8 assay. CHO-K1 cell proliferation was evaluated by plating 5 × 10^3^ cells per well in 96-well plates. After 24 hours in serum-containing DMEM-F12 medium, the cells were incubated with a serum-free DMEM-F12 solution of unlabeled or dye-labeled peptides for 2 hours at 37 °C. After the incubation, the medium was removed, cells were washed with PBS 1X and incubated for 2 hours at 37 °C with 100 μl of a 10%-solution of WST-8 dissolved in serum-containing DMEM-F12. Absorbance at 450 nm was measured using a multiplate reader (Microplate Reader, GloMax®-Discover and Explorer Systems, Promega). Cell viability was quantitatively determined by comparing peptide-treated cells with untreated cells (as a reference of 100% viability) and cells treated with dimethyl sulfoxide (DMSO) 20% v/v. Reported data represent the average of three independent experiments, in which three wells for each concentration were measured. Error bars represent the standard errors.

### Critical Micelle Concentration (CMC) determination

All the peptide solutions were always freshly prepared and dye-labeled peptides were quantified by UV-Vis absorbance. Unlabeled peptides were quantified by amino acidic digestion obtained by the mineralization of a known amount of peptide (5–50 μg) dissolved in 200–500 μl of HCl 6 M, with the following conditions: temperature: 170 °C; time: 20 minutes. Finally, derivatized amino acids were detected by RP-HPLC (Jupiter 4 μm Proteo 90 Å column (250 × 4.6 mm; Phenomenex) with these solvents: water:TFA 100:0.01 v/v (eluent A)/acetonitrile:water:TFA 95:5:0.01 v/v (eluent B), flux 1 ml/min, and their identity was confirmed by electrospray mass spectroscopy. CMC values of peptide and labeled-peptide systems were then determined by the *pyrene 1:3 ratio* method^15^. Fluorescence emission spectra at 25 °C of unlabeled and labeled peptide water solutions containing 5 × 10^−7^ μM pyrene were recorded using an excitation wavelength of 335 nm. The intensities I_1_ and I_3_ were measured at the wavelengths corresponding to the first and third vibronic bands of pyrene (373 nm, emission range: 368–378 nm, and 385 nm, emission range: 380–390 nm, respectively). According to literature^15^, the plot of the *pyrene 1:3 ratio* index as a function of the concentration of the surfactant can be described by a decreasing sigmoid of the Boltzmann type and indicates the transition from the monomeric to the aggregated form of the peptide. Experimental raw data were fitted using the OriginPro8 software, and the center of the sigmoid is identified as the characteristic CMC.

### Dynamic Light Scattering (DLS) measurements

DLS measurements were performed at 25 °C in a 50-μL quartz cuvette on a Zetasizernano ZS DLS (Malvern Instrument) following the manufacturer’s instructions. Water solutions of labeled-peptide at 100 μM were analyzed with a single scattering angle of 90°. Each value reported is the average of five consecutive measurements.

## Additional Information

**How to cite this article**: Macchi, S. *et al*. Spontaneous membrane-translocating peptides: influence of peptide self-aggregation and cargo polarity. *Sci. Rep*. **5**, 16914; doi: 10.1038/srep16914 (2015).

## Supplementary Material

Supplementary Information

Supplementary Movie S1

Supplementary Movie S2

Supplementary Movie S3

## Figures and Tables

**Table 1:** Critical Micelle Concentration (CMC) from pyrene 1:3 ratio method and hydrodynamic radius (R_H_) from DLS for unlabeled and dye-labeled TM9 and TP2 peptides.

Peptide	TM9	TM9-ATTO 425	TM9-TAMRA	TP2	TP2-ATTO 425	TP2-TAMRA
CMC (μM)	58 ± 4	5.7 ± 0.8	3.8 ± 0.5	1.0 ± 0.2	7.1 ± 0.9	3.7 ± 0.5
R_H_ (nm)	112 ± 14	156 ± 8	135 ± 14	163 ± 6	114 ± 11	113 ± 12
