# Supplementary material for: Spontaneous membrane-translocating peptides: influence of peptide self-aggregation and cargo polarity
Source: Sci Rep. 2015 Nov 16;5:16914. doi: 10.1038/srep16914 (PMC4645181; doi:10.1038/srep16914)
Supplement: Supplementary Information [file srep16914-s1.doc]

**Supporting Information**

**Spontaneous membrane-translocating peptides: influence of peptide self-aggregation and cargo polarity**

Sara Macchi^a^, Giovanni Signore^b^, Claudia Boccardi^b^, Carmine Di Rienzo^a,b^, Fabio Beltram^a,b^, and Francesco Cardarelli^b,*^

^a^ NEST, Scuola Normale Superiore and Istituto Nanoscienze-CNR, Piazza San Silvestro 12 - 56127 Pisa, Italy

^b^ Center for Nanotechnology Innovation @NEST, Istituto Italiano di Tecnologia, Piazza San Silvestro 12 - 56127 Pisa, Italy

^*^ To whom correspondence should be addressed: [francesco.cardarelli@iit.it](mailto:francesco.cardarelli@iit.it)


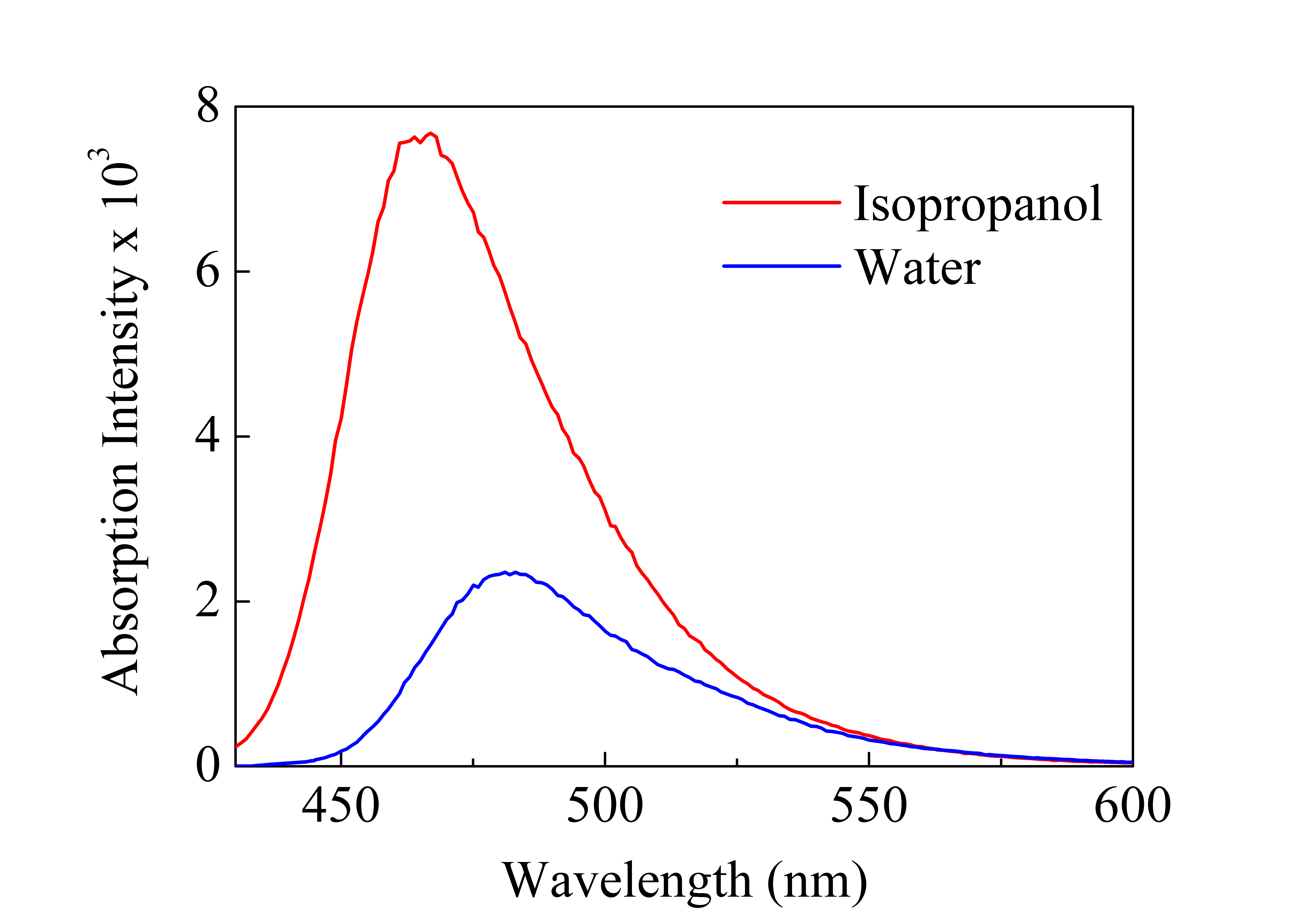


**Fig S1:** The desired amount of ATTO 425 was dissolved in isopropanol or in water, and the solutions were analyzed by UV-Vis absorbance. Absorption spectra of ATTO 425 fluorophore in isopropanol (dielectric constant: 18) and in water (dielectric constant: 80) with λ_exc_ = 436 nm are shown. A red shift is present passing from the former to the latter, indicating that this coumarin structured fluorophore has solvatochromic properties. As the dielectric constant of the lipophilic membranes is very similar to the isopropanol value, the imaging contrast between the membrane enriched cell and the aqueous external solution is very high.


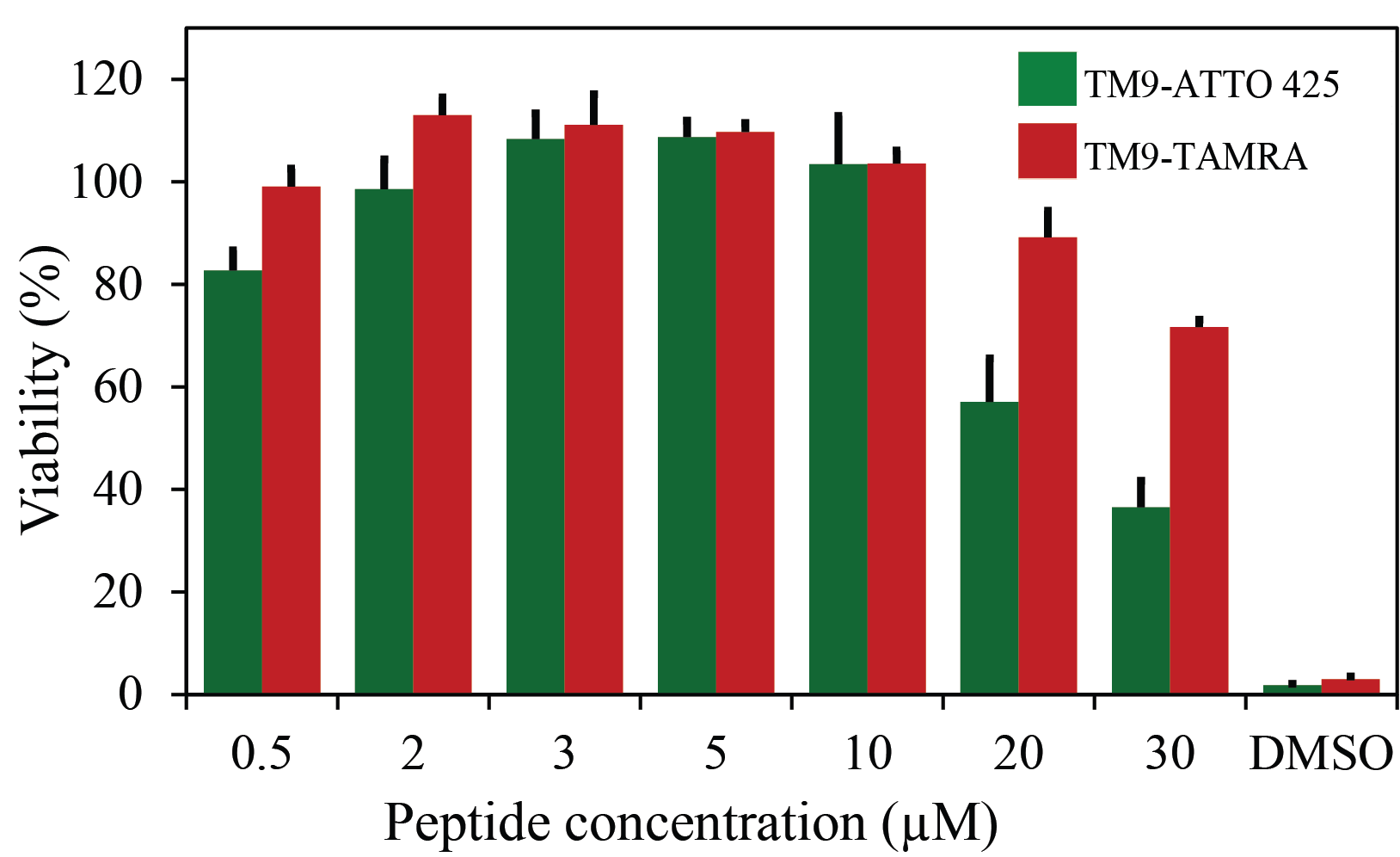


**Fig S2:** Cell metabolic activity of TM9-ATTO 425 and TM9-TAMRA measured with WST-8 assay. In general, above 10 µM peptide concentration, a decrease in cell viability is detected. This effect is more pronounced for TM9-ATTO 425 (i.e. viability decreases to 60% and 40% at 20 µM and 30 µM, respectively) as compared with cells treated with the same concentrations of TM9-TAMRA (i.e. viability decreases to 90% and 70%, respectively). This prompts us to speculate that an additional effect on viability might be caused by peptide redistribution within cytoplasm. Here untreated cells are defined as 100% viable (not shown), while cells exposed to 20% dimethyl sulfoxide (DMSO) are used as positive control for a decreased metabolic activity.

**Fig S3:** Critical Micelle Concentration by pyrene 1:3 ratio method for fluorophore solutions in water. For TAMRA the characteristic CMC is 80 ± 14 µM, while for ATTO 425 it is 25.7 ± 4.1 µM.

**Fig S4:** Labeling of GAGs with WFA-FITC in CHO-K1 cells before (on the left) and four hours after (on the right) the administration of ChABC (final concentration: 0.2 U/ml). Arrows indicate cell surface labeled GAGs. Scale bars: 10 µm.


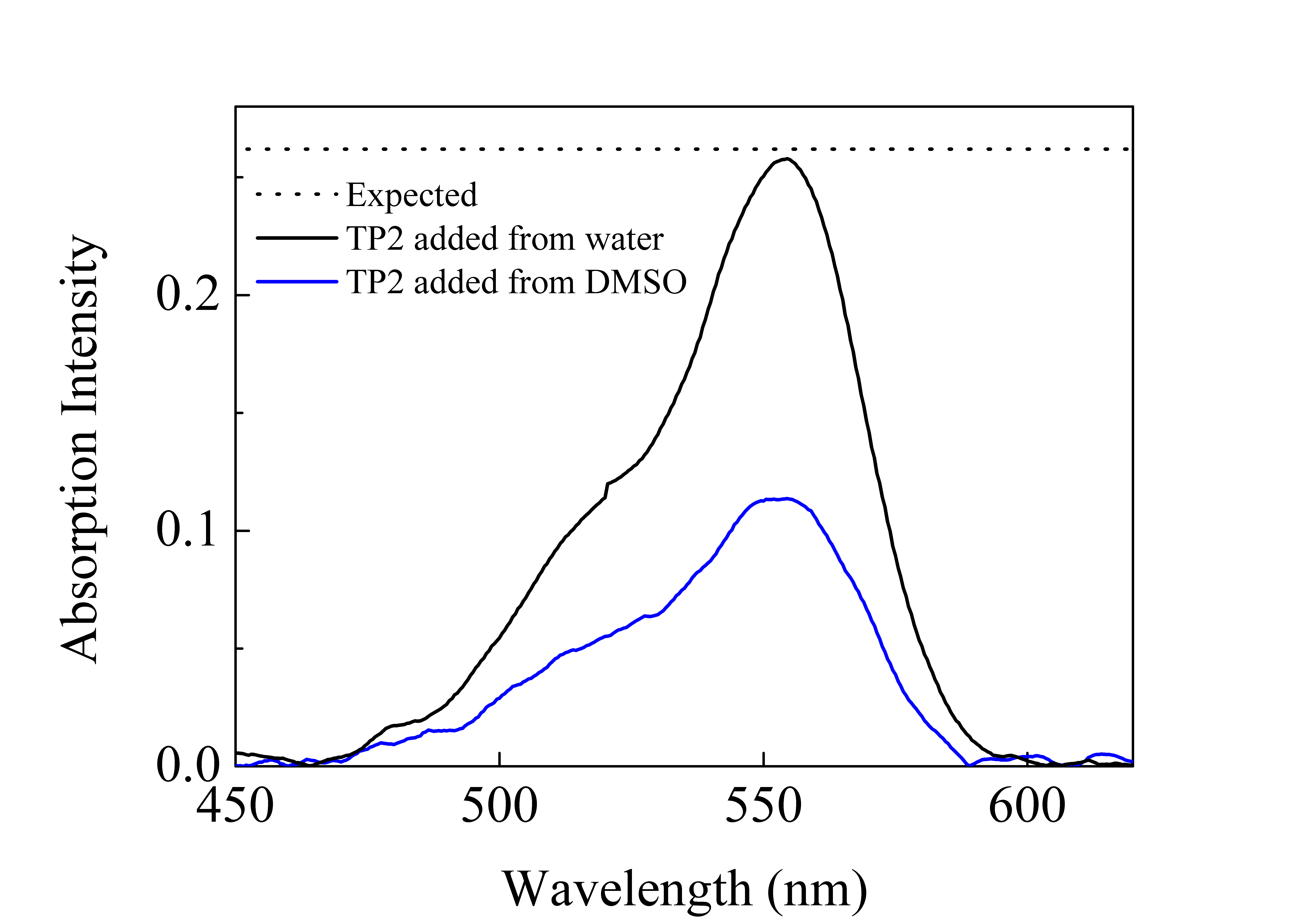


**Fig S5:** UV absorption spectra of TP2 at 2μM concentration in water, measured upon addition of the peptide directly from DMSO stock solution (blue trace) or from a water solution (black trace). Only this latter procedure yields the expected absorption spectrum (indicated by the dashed black line). This experiment shows that nearly 60% of the peptide is apparently lost upon direct addition from DMSO to water. This in turn is consistent with the formation of a stabilized phase that is not diffusing in solution, but likely under the form of a precipitate.

**Movie1:** TM9-ATTO 425 translocation starting from the Nucleation Zones in CHO cells at 37°C and with 5% CO_2_. Dye-labeled peptide concentration: 12 µM in no serum growth medium. TM9-ATTO 425 shows massive cytoplasmic staining in about 50 % of the cells. Scale bar: 10µm. Movie acquisition starts about 60 seconds after the addition of the peptide and both fluorescent and bright field images are recorded every 5 s. Field of view = 246 x 246 µm.

**Movie2:** TM9-ATTO 425 homogeneous translocation in PgsA-745 cells at 37°C and with 5% CO_2_. Dye-labeled peptide concentration: 10 µM in no serum growth medium. TM9-ATTO 425 shows massive cytoplasmic staining in almost all the cells. Scale bar: 10µm. Movie acquisition starts about 60 seconds after the addition of the peptide and both fluorescent and bright field images are recorded every 10 s. Field of view = 174 x 174µm.

**Movie3:** TM9-ATTO 425 homogeneous translocation in CHO cells at 37°C and with 5% CO_2_. Dye-labeled peptide concentration: 10 µM in no serum growth medium. TM9-ATTO 425 shows massive cytoplasmic staining in almost all the cells. Scale bar: 10µm. ChABC (final concentration: 0.2 U/ml) for proteoglycans digestion is administered four hours before we recorded the movie. Movie acquisition starts about 60 seconds after the addition of the peptide and both fluorescent and bright field images are recorded every 13 s. Field of view = 81.4 x 81.4µm.
